# Water Permeates and Plasticizes Amorphous Carbon Dots: Unraveling the Inner Accessibility of the Nanoparticles by Glass Transition Studies

**DOI:** 10.1002/adma.202510992

**Published:** 2025-09-22

**Authors:** Elisa Sturabotti, Valerio Di Lisio, Lucia Cardo, Alessandro Camilli, Estela Moretón Alfonsín, Daniele Cangialosi, Amaia Iturrospe Ibarra, Arantxa Arbe, Maurizio Prato

**Affiliations:** ^1^ Center for Cooperative Research in Biomaterials (CIC biomaGUNE) Basque Research and Technology Alliance (BRTA) Donostia – San Sebastián 20014 Spain; ^2^ Donostia International Physics Center (DIPC) Paseo Manuel de Lardizabal 4 Donostia – San Sebastián 20018 Spain; ^3^ Centro de Fisica de Materiales (CFM‐MPC) CSIC‐UPV/EHU Paseo Manuel de Lardizabal 5 Donostia ‐ San Sebastián E‐20018 Spain; ^4^ Ikerbasque Basque Foundation for Science Bilbao 48013 Spain; ^5^ Department of Chemical and Pharmaceutical Sciences, INSTM UdR Trieste University of Trieste Trieste 34127 Italy

**Keywords:** carbon dots, carbon nanodots, glass transition, plasticizers, structure‐property relationship, water diffusion

## Abstract

Carbon dots (CDs) are highly versatile nanomaterials with promising applications across catalysis, sensing, biotechnology, and optoelectronics. Yet, despite the widespread interest, their internal structure and permeability to solvents remain poorly understood. In this study, novel insights are provided into the plasticizing effect of water on amorphous CDs, addressing a critical gap in the current knowledge. A new class of polyamide‐based CDs is synthesized via a microwave‐assisted reaction using an amino acid and a small polyamine in water, systematically varying reaction times (3 – 60 min) to tailor the compactness of the carbon core. These findings reveal that water can permeate the internal structure of CDs and act as a plasticizer, significantly lowering their glass transition temperature, as shown by calorimetric analyses. Less compact CDs absorbed more water, while denser ones showed lower permeability. These trends are further corroborated by X‐ray scattering data. This report clearly demonstrates that the carbonaceous core of CDs is accessible to water, challenging the assumption that the nanoparticles are structurally impermeable. These results lay the groundwork for new structural models of CDs and open exciting opportunities for diffusion studies, functional design, and targeted applications in nanoscience.

## Graphical abstract

The water permeability of amorphous carbon dots (CDs) is demonstrated by investigating their plasticization. Novel polyamide‐based and amorphous nanoparticles are synthesized by controlling their inner packing density. Water plasticization is evidenced by the decrease of the CDs glass transition temperature with increasing the hydration degree. More compact CDs structure resulted in lower water uptake.

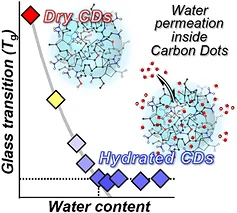

## 1 Introduction

Porous and entangled nanomaterials can absorb defined amounts of dispersant media within their confined internal structures. Consequently, liquid diffusion through ultrasmall architectures has both theoretical significance and practical implications. When water is intercalated into such nanostructures, it can induce unusual dynamics, so that studying these phenomena may help reveal new and unexpected physicochemical properties.^[^ ^1^, ^2^ ^]^ Moreover, the local hydration state of biomolecules—such as proteins, DNA, enzymes, or nanozymes—plays a critical role in determining their structural integrity and biological functionality.^[^ ^3^, ^4^, ^5^, ^6^, ^7^ ^]^ Similar dependencies are also observed in organic and inorganic catalysts, sensors, and metal‐organic frameworks.^[^ ^8^, ^9^ ^]^ Notably, water diffusion can serve as a valuable probe for assessing the swelling capacity of confined systems.^[^ ^10^, ^11^ ^]^ This allows for the exploration of their “expandable” nature and internal accessibility at the nanoscale, depending on factors such as conformational rearrangements, hydrophilic/hydrophobic character, and the degree of chain cross‐linking, branching, or entanglement within the nanoparticles.^[^ ^12^ ^]^

In this context, we investigated water diffusion in carbon dots (CDs, also referred to as carbon nanodots, CNDs), a class of ultrasmall carbon‐based nanoparticles whose true structure remains under active debate. Since their discovery in 2004,^[^ ^13^ ^]^ CDs have attracted broad scientific interest due to their applicability in fields such as catalysis,^[^ ^14^ ^]^ optoelectronics,^[^ ^15^ ^]^ biotechnology,^[^ ^16^ ^]^ nanomedicine,^[^ ^17^, ^18^ ^]^ and sensing.^[^ ^19^ ^]^

Although certain issues regarding their synthesis and purification remain unresolved,^[^ ^18^, ^20^, ^21^ ^]^ it is accepted that CDs consist of a dense, often polymer‐like inner core surrounded by surface functional groups. The core is generally described as hydrophobic and “inaccessible”, being highly dehydrated and composed of either amorphous, randomly cross‐linked carbon or partially ordered sp^2^ domains (graphitic CDs).^[^ ^22^ ^]^ This part of the CDs is considered primarily responsible for their photocatalytic activity.^[^ ^23^, ^24^ ^]^ The surface, in contrast, is enriched with hydrophilic polymeric chains bearing functional groups—amines, carboxylic acids, hydroxyls, etc.—that facilitate solubility in various solvents, post‐synthetic modifications,^[^ ^25^ ^]^ and catalytic activity.^[^ ^26^ ^]^

While surface chemistry is well‐characterized, the accessibility of the CDs core to analytes and solvents remains largely unexplored. To date, dynamic simulations carried out on highly crystalline CDs—composed of overlaid ordered graphene‐like planes—have proven that the dots are stabilized in aqueous or organic solution by an extensive hydrogen bond network formed between the surrounding solvent molecules and the superficial functional groups (hydroxyls, carbonyls or neutral and charged carboxyl moieties), thus confirming the shell role in the colloidal stability.^[^ ^27^ ^]^ Anyway, no solvent penetration inside the nanostructure was accounted for in the molecular dynamics although the role of the core is central for many CDs activities.

For example, in enzyme‐mimicking applications (nanozymes), the catalytic activity—oxidase, peroxidase, or reductase‐like—relies heavily on the proximity between the nano‐catalyst and the reactants, whether the catalysis is mediated by undoped CDs or by metal‐doped variants.^[^ ^28^, ^29^ ^]^ Similarly, the water uptake in paramagnetic metal‐ion‐doped CDs (e.g., Gd(III),^[^ ^30^ ^]^ Mn(II),^[^ ^31^ ^]^ or Fe(III)^[^ ^32^ ^]^), largely investigatedfor Magnetic Resonance Imaging (MRI) applications, affects both signal strength and relaxation times, being the two related to the accessibility of the coordination environment.^[^ ^18^, ^33^ ^]^ Therefore, the understanding of water diffusion into CDs is crucial to probe their internal architecture and achieve a more robust knowledge of their nature. To this aim, synergistic thermodynamic and structural characterization of CDs in aqueous environment may shed light onto solvent permeation phenomenology.

The thermodynamic state of any system—described by macroscopic variables like volume, internal energy, and enthalpy—can be significantly altered by the incorporation of an additional component. In amorphous materials, the diffusion of a substance like water can modify these thermodynamic variables, leading to detectable changes in critical phase transition temperatures, most notably the glass transition.^[^ ^34^ ^]^ The latter, defined by the glass transition temperature (*T*_g_), marks a fundamental kinetic transformation from a rigid, disordered solid (glass state) to a more mobile, viscous state (rubber or liquid state).^[^ ^35^ ^]^ This process is triggered when structural units—molecules, polymer segments,^[^ ^36^, ^37^ ^]^ or atoms^[^ ^38^, ^39^ ^]^—gain sufficient mobility for translational motion. Accompanying this transition are abrupt changes in viscosity, heat capacity, and mechanical properties.

*T*_g_ serves as a highly sensitive indicator of structural flexibility, and its modulation—via water diffusion or small molecule intercalation—can reveal important insights into material behavior.^[^ ^40^ ^]^ When a low molecular weight substance migrates through a polymer amorphous matrix, it acts as a plasticizer, increasing the flexibility of polymeric chains and thus significantly lowering the *T_g_*.^[^ ^41^ ^]^ This phenomenon, known as plasticization, has been studied for decades in amorphous and semi‐crystalline polymers.^[^ ^42^ ^]^

Specifically, the water diffusion through hydrophilic polymers, such as the class of polyamides, has been the subject of extensive research.^[^ ^43^ ^]^ These studies have provided a comprehensive understanding of the relationship between the acceleration of polymeric relaxation mechanisms and water diffusion and uptake.^[^ ^44^ ^]^ In biological systems, water plasticization is ubiquitous and has been encountered in numerous biomolecules, such as small globular proteins. Significant *T_g_* depressions as a consequence of hydration has been described, among the others, for lysozyme,^[^ ^3^ ^]^ wheat gluten proteins,^[^ ^45^ ^]^ Bovine Serum Albumin (BSA)^[^ ^46^ ^]^ and whey proteins.^[^ ^47^ ^]^ Although a universal trend can be hard to define, the major effect of hydration on the proteins’ *T_g_* was observed for water contents close to 20 – 40 wt.% and it was less pronounced for the more constrained proteins. Furthermore, calorimetric investigations of protein/water mixtures detected the existence of strongly interacting water, intercalated in the inner structure. This water type, “buried” in the protein core or close to the heteroatoms in the structure, acts as a plasticizer and enables protein conformational dynamics, thus having a starring role in some protein elasticity properties, folding capabilities, enzymatic activity and functionalities.^[^ ^48^ ^]^

Giving this context, we present here an investigation on the water diffusion in amorphous polymer‐based CDs by tracking changes in their *T*_g_. To the best of our knowledge, no comprehensive calorimetric studies have been conducted to directly assess water permeability in CDs. Moreover, the glass transition itself has rarely been associated with these nanomaterials, despite their polymeric nature.^[^ ^49^, ^50^, ^51^ ^]^

We synthesized a series of novel polyamide‐based CDs using β‐Alanine (β‐Ala) and triethylenetetramine (TETA) as precursors. The amorphous and hydrophilic character of the nanoparticles, as confirmed by broad diffraction peaks and observable glass transitions, provided an ideal system for exploring water interactions with the carbonaceous network. Using Differential Scanning Calorimetry (DSC), including Fast Scanning Calorimetry (FSC), we quantified the *T*_g_ shifts associated with water uptake of the nanoparticles. CDs synthesized with varying reaction times exhibited different *T*_g_ values in the dry state and distinct swelling behaviors, depending on structural rigidity.

Small‐Angle X‐ray Scattering (SAXS) further revealed that internal nanoparticle compactness in aqueous suspensions varied significantly: short reaction times produced loosely packed and expanded structures, whereas longer times yielded globular, more compact morphologies. These differences directly influenced water uptake capacity.

Altogether, our findings challenge the prevailing view of CDs as inaccessible, dehydrated materials. Instead, we show that CDs can absorb and retain defined amounts of water, modulated by their internal compactness, having a structure that resembles cross‐linked or branched polymeric ultrasmall nanoparticles. This concept opens new avenues for understanding CDs' structural behavior and for developing future applications in catalysis, sensing, and molecular diffusion.

## 2 Results and Discussion

**Figure 1:**
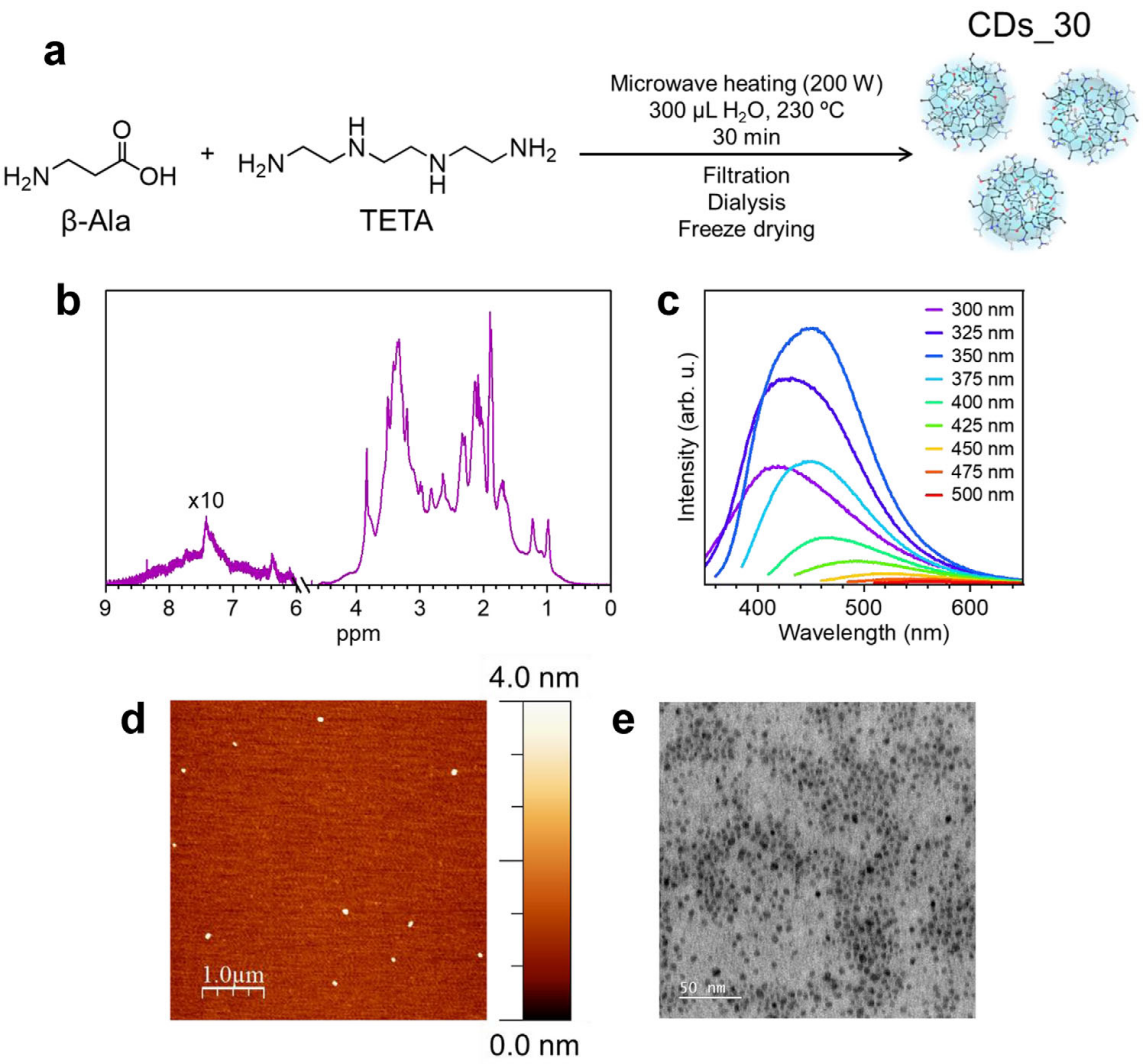
Synthesis and characterization of the carbon dots obtained after 30 min of reaction (CDs_30). a) Reaction scheme: β‐Alanine (β‐Ala) and triethylenetetramine (TETA) are reacted in Milli‐Q water using a microwave assisted approach. After the reaction, the crude nanoparticles are filtered (0.2 µm), dialyzed (MWCO = 0.5 – 1 kDa) and freeze dried to obtain a brown and hygroscopic powder. Fluorescent and entangled CDs are sketched at the end of the reaction. b) ^1^H NMR profile of CDs recorded in D_2_O at 500 MHz. Aliphatic and amidic zones are presented with different magnifications. c) CDs fluorescence spectra recorded in water at 0.1 mg mL^−1^. d) AFM and e) TEM images of CDs_30 at concentration of ng mL^−1^.

Blue‐emitting carbon dots (CDs) were synthesized in water using a microwave‐assisted approach. Although the precise reaction pathways leading to CD formation are still not fully understood,^[^ ^52^, ^53^ ^]^ the choice of suitable and chemically compatible precursors is crucial for controlling the properties of the resulting nanomaterials. This approach helps avoid misleading results or speculative interpretations of the reaction outcomes. In this work, β‐Alanine (β‐Ala) and triethylenetetramine (TETA), an amino acid and a small molecular weight polyamine respectively, were selected for the preparation of novel amine‐containing CDs. The synthetic parameters have been specifically set to obtain positively charged nanoparticles, useful as versatile platform for biomedical application, e.g., drug grafting, sensing, antibacterial, or antimycotic applications.^[^ ^54^, ^55^, ^56^, ^57^ ^]^ Due to the nature of the starting materials, we expected the CDs to possess a nanostructure mainly composed of disordered, i.e., amorphous, and hydrophilic polyamide chains, covalently cross‐linked with each other, and surrounded by an amino‐functionalized surface. The optimization and first attempts to obtain CDs are summarized and commented in Table S1 (Supporting Information). As initial screening, ^1^H NMR (Figure S1, Supporting Information) was used to characterize the reaction products and to verify the plausible nanoparticles formation. Indeed, while small molecular weight molecules show sharp peaks with precise and recurring multiplicity, the CDs profiles are characterized by broad peaks along the proton spectrum. This behavior depends on the different magnetic environments and slow rotations that protons entrapped in complex nanostructures encounter, thus resulting in enlarged magnetic signals.^[^ ^58^ ^]^ The thorough analysis of CD NMR spectra is therefore becoming essential in the CD field, to evince whether a reaction is successful and/or it is necessary to implement additional purifications for molecular fluorophores' removal.^[^ ^18^, ^59^ ^]^ Among others, CDs_30 (reaction scheme in **Figure** **1**a, 30 stands for 30 min of reaction time) presented the best NMR profile (Figure 1b), with broadened proton signals centered in the aliphatic region of the spectrum and low intensity signals between 6 and 9 ppm, ascribable to amidic protons. CDs_30 displayed blue centered emission (Figure 1c) and, as confirmed by AFM and TEM, the quasi‐spherical nanoparticles exhibited homogeneous shape (Figure 1d,e). CDs_30 possess average height of 3.2 ± 1.3 nm and a mean diameter of 4.0 ± 1.3 nm, as measured by AFM and TEM analysis respectively (detailed images and sizes distribution for sample CDs_30 are reported in Figures S5–S8, Supporting Information).

**Figure 2:**
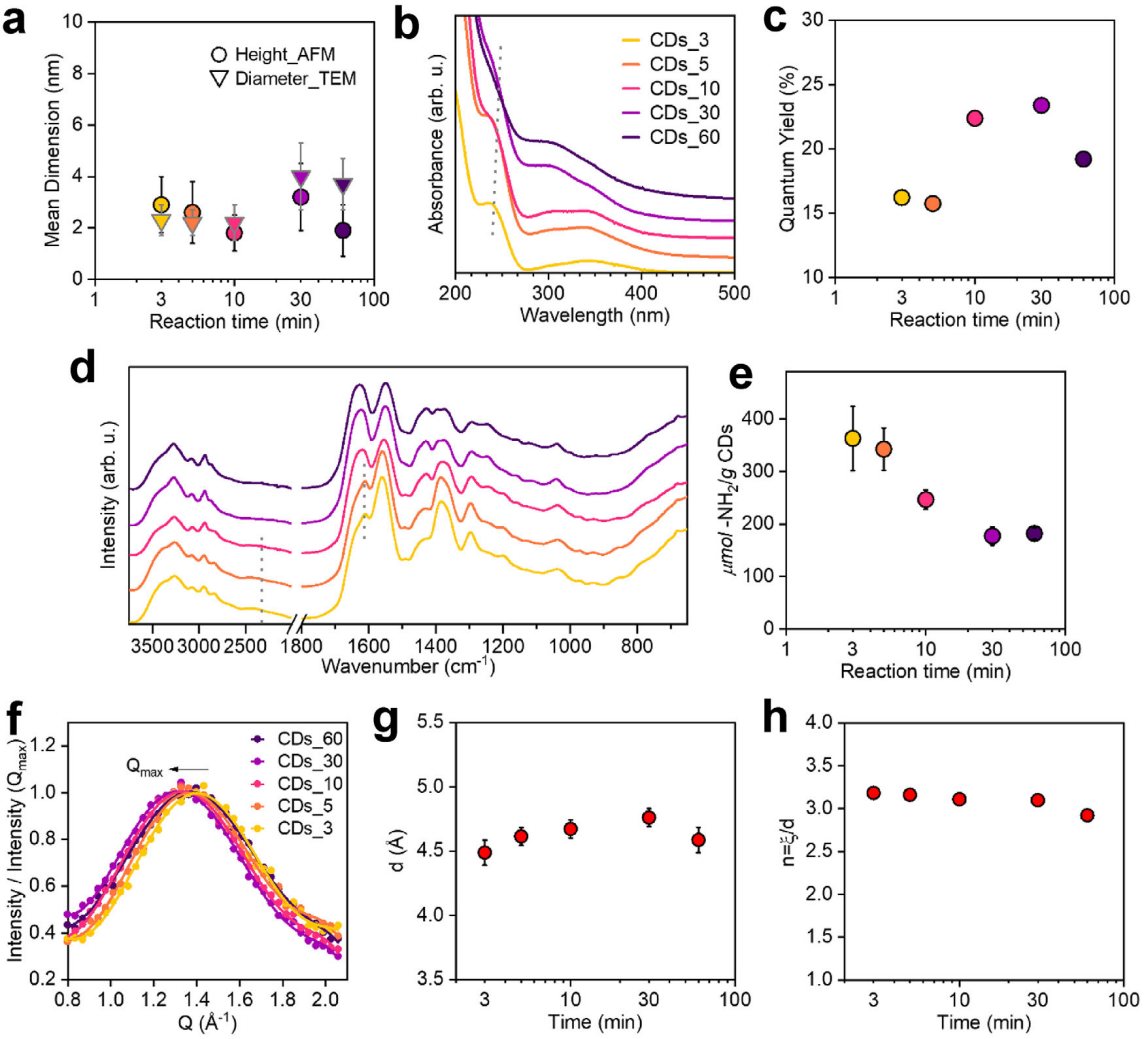
Comparison of features for CDs obtained after 3 (yellow), 5 (orange), 10 (pink), 30 (light purple), and 60 (dark purple) minutes of reaction. a) AFM and TEM average dimension for each time point. b) UV–vis spectra of CDs at 0.1 mg mL^−1^ in Milli‐Q water. c) Quantum yield percentage values measured at 350 nm. d) ATR‐FTIR spectra of dried samples. e) Ninhydrin assay results for the quantification of reactive ‐NH_2_ moieties. f) WAXS results (structure factors) on bulk samples of dried CDs. Lines are guides for the eye. g) Average intermolecular distance for dry samples. h) Ratio between the short‐range order correlation length and the intermolecular distance retrieved for dry samples.

Starting from CDs_30, we investigated the time/structural dependence of CDs by changing reaction time (3, 5, 10, 30, and 60 min) and keeping the β‐Ala/TETA molar ratio constant (Table S2, Supporting Information). Again, ^1^H NMR profiles were preliminary analyzed for each set of dots (Figure S3, Supporting Information). At each time point, the spectra were characterized by broad proton signals, compatible with the presence of nanoparticles. The resulting mass yield percentage was quite low for the shorter reactions, reasonably because of a partial carbonization process, while for longer times good yields were achieved with good reproducibility. The formation of nanoparticles was confirmed again by AFM and TEM studies, as shown in Figures S5 and S7 (Supporting Information). CDs prepared at different timepoints were characterized by well‐dispersed particles with good homogeneity in terms of sizes and shapes (AFM and TEM sizes in **Figure** **2**a; for detailed images and sizes distribution of each sample see Figures S5–S8, Supporting Information). No large aggregates were observed, while the particles dispersed well on the mica and Lacey carbon film.

The spectroscopic properties of the nanoparticles showed a similar optical trend for all the samples tested. The UV‐vis spectra (Figure 2b) presented three main peaks centered at 240, 310, and 350 nm, corresponding to σ→σ* and π→π* for double bonded carbons or to *n*→π* for doubly bonded heteroatoms.^[^ ^60^ ^]^ Interestingly, the peak intensity changed with reaction times and the 240 nm signal (dotted gray line) almost disappeared with the formation of a more coordinated carbonaceous structure and the loss of molecular‐like structures (bathochromic shift).^[^ ^61^ ^]^ Reaction times did not strongly affect the CD fluorescence trend (Figure S9, Supporting Information) and all the samples exhibited the typical wavelength‐dependent profile. The blue luminescence, extended between 350 and 650 nm, displayed a maximum of emission centered at 460 nm when CDs were excited at 350 nm. As reported by Vallan and coauthors, blue emission is often common for polymeric and polyamide‐based carbon dots.^[^ ^62^ ^]^ Indeed, the photoluminescence of entangled and nonconjugated polyamide CDs is caused by the decrease of vibrational and rotational freedom of sub‐fluorophores units, such as the C═O, C═N and other heteroatom‐containing double bonds, which facilitates their radiative relaxation (cross‐link enhanced emission—CEE—effect). A slight increase in the QY % was evidenced with reaction times, with values ranging from 16% to 24% (Figure 2c; and Table S3, Supporting Information), reasonably due to the increase on nanostructure packing for longer reaction times (vide infra). This effect seems to be counterbalanced by the decrease in nitrogen content (see XPS results in Table S4, Supporting Information) that possibly reduces the nanoparticles luminescence at longer reaction times.^[^ ^63^ ^]^ In addition to optical aspects, the chemical composition of CDs is fundamental for further exploring their structural evolutions with time, hence FTIR and XPS spectroscopies were performed. In the ATR‐FTIR spectra (Figure 2d), the two broad signals at 3430 and 3270 cm^−1^ correspond to the stretching of ‐NH‐ moieties while the band at 2940 cm^−1^ is associated to the symmetric and asymmetric stretching of the aliphatic components in the structure. Interestingly, infrared resonance of ammonium groups at 2400 cm^−1^, as well as the NH bending of aminic moieties at 1610 cm^−1^, is lightly visible for CDs_3 and CDs_5 (gray dashed lines), reasonably due to the abundance of more and free aminic‐based functionalities at lower reaction times. The condensation between an amino acid and a mono‐ or polyamine, as for our starting reagents, leads to amidic bonds formation. This mechanism can be followed by analyzing the spectral changes occurring at the expense of the carboxyl and amidic moieties. In fact, the two peaks at 1570 and at 1380 cm^−1^, ascribable to carboxylate asymmetric and symmetric stretching, are visible only for samples CDs_3 and CDs_5 and disappear with increasing time. Instead, the two new bands at 1630 and 1550 cm^−1^, associated with carbonyl stretching (amide I) and ─NH bending (amide II), increase in magnitude. As the reaction proceeds, changes were mainly visualized in the amide I/amide II relative band intensities, confirming the evolution of the CD structure for longer reaction times. Lastly, the peaks at 1430, 1240, and 1040 cm^−1^ were assigned to the infrared absorption of the CD organic framework, such as the ‐CH bending, ‐CN stretching and other interchain vibrations. The presence of carbon, nitrogen and oxygen in all the CDs was corroborated by XPS spectroscopy (Figure S10 and Table S4, Supporting Information). In particular, the C 1s signal, between 282 and 292 eV, was composed of four components associated to C─C/C═C at 284.8 eV, C─O/C─N at 285.9 eV, C═O/C═N at 287.5 eV and O─C═O at 288.8 eV, confirming the variety of heteroatomic functionalities. Notably, the amount of double bonded moieties increased moving from 3 to 60 min of reaction. The N 1s signal (394 − 406 eV) was mainly composed of aminic and ammonium components at 399.5 and 401.0 eV, respectively. The ammonium contribution decreased with reaction times, confirming along with the FTIR the consumption of free and protonable aminic groups with the progression of polyamide network formation. The decrease in reactive amine content, from ≈350 to 170 µmol of ‐NH_2_ per g of CDs, was also corroborated by the colorimetric ninhydrin assay (Figure 2e). Lastly, the O 1s signal at 526 – 538 eV was deconvoluted into two main components, one at 531 eV, referred to C═O, and another at ≈ 532 eV, associated with C─O.

To retrieve structural features of our polyamide‐based CDs, we exploited the information offered by X‐ray diffraction techniques, in particular to the scattering vector (Q) dependence of the measured intensities. In contraposition to the sharp Bragg peaks corresponding to crystalline solids—as those presented e.g., by the β‐Ala, see Figure S13a—the diffraction patterns of the dry bulk samples obtained by Wide Angle X‐ray Scattering (WAXS) display a broad main peak (Figure 2f). The finite size of crystalline nanoparticles leads to broadening of Bragg reflections;^[^ ^64^ ^]^ this effect can be exploited, in fact, to estimate the size of such nano‐objects from the width of the peak using in a first approximation the Scherrer law.^[^ ^65^, ^66^ ^]^ From such an approach, nanoparticle diameters of the order of 7 Å would be deduced for the CDs, which are clearly incompatible with the sizes deduced from AFM/TEM (and, as will be shown below, from SAXS experiments). The WAXS patterns are by far much broader than expected from finite size effects and, actually, look very similar to those typical for amorphous systems, where the main peak—so‐called ‘amorphous halo’—reflects the short‐range order in the system, in particular correlations between pairs of atoms belonging to different molecules or chain segments. From its position (*Q_max_*), the average inter‐molecular distance *d* can be deduced in the Bragg approximation as *d* = *2π*/*Q_max_*, taking values ≈4.6 Å (see Figure 2g). On the other hand, the width of the amorphous halo is a measure for the short‐range order correlation length *ξ* of the monomers, which relates to the distribution of chain distances around the mean value. Within the uncertainties, this magnitude (estimated as *ξ* = *4π*/FWHM, with FWHM the peak width at half maximum) is of ≈14 Å independently of the reaction time (Figure S14, Supporting Information marked as dry_CDs). This implies that the correlation extends over ≈3 intermolecular distances (see *n* = *ξ*/*d* in Figure 2h). Compared with simple linear polymers in bulk as e.g., polybutadiene (*d* ≈ 4.2 Å, *ξ* ≈ 34 Å, *n* ≈ 8)^[^ ^67^ ^]^ or polyisobutylene (*d* ≈ 6.4 Å, *ξ* ≈ 30 Å, *n* ≈ 5)^[^ ^68^ ^]^ the correlation length is smaller and the short‐range order extends over less intermolecular distances. In fact, the results are similar to those in the pure TETA precursor at room temperature, which is in the liquid state (Figure S13b, Supporting Information). Thus, WAXS results undoubtedly evidence the amorphous character of the molecular arrangements within the dry CDs.

Considering the structural pieces of evidence discussed above, some physicochemical characteristics of the amorphous phases would be expected, including the signatures of the glass transition. The characteristic *T*_g_ value of a material depends upon various factors, one of which is its chemical composition. Since our CDs are polyamide‐based materials, it is plausible that the *T*_g_ of bulk particles falls within the typical temperature range of other known polyamides. Thanks to strong intra‐ and intermolecular hydrogen bonding within amidic moieties, structural rigidity, and chains entanglement, polyamides show relatively high *T*_g_, way above room temperature (between 40 and 140 °C).^[^ ^69^ ^]^

**Figure 3:**
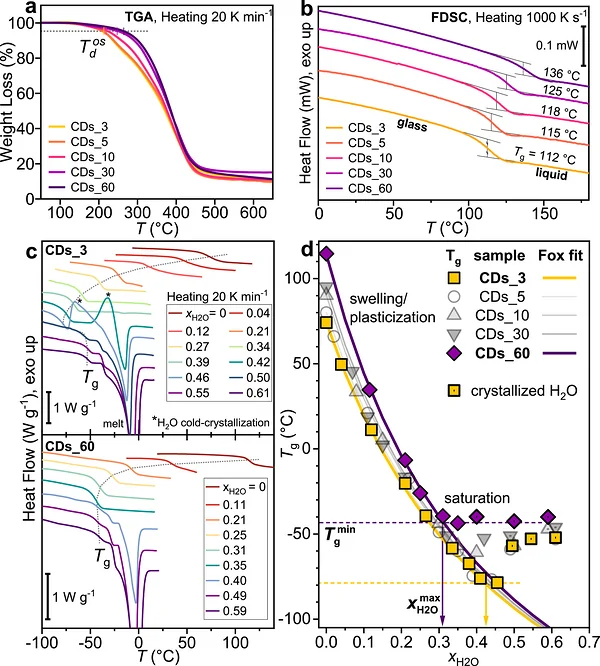
Thermal characterization of CDs. a) TGA curves of CDs in N_2_ atmosphere. The degradation temperature onset (*T*_d_^os^) is taken when the sample weights are 95% of the initial mass. b) FDSC heating curves (vertically shifted), acquired after drying the CDs at 180 °C for 1 min. *T*_g_ is determined at the mid‐point of the heat flow step. c) DSC heating thermograms of hydrated CDs_3 and CDs_60 at different water weight fraction *x*_H2O_. The thermograms were vertically shifted for clarity. The dashed gray line is a guide to the eyes to better visualize the *T*_g_ displacement. d) *T*_g_ variation as a function of *x*_H2O_ for the overall CDs series. The graphical determination of *x*_H2O_^max^ is exemplified for CDs_3 and CDs_60.

To retrieve some thermal features of the dry CDs, we explored their thermal behavior by Thermogravimetric Analysis (TGA), as well as Flash Differential Scanning Calorimetry (FDSC). TGA (**Figure** **3**a) was carried out to detect the region of stability of our nanomaterials, prior to the calorimetric characterization. The main degradation, marked by the degradation temperature (*T*_d_), occurs at ≈390 ± 5 °C for all the specimens, meanwhile the degradation onset (*T*_d_^os^) shifts from 215 to 275 °C, as the CDs reaction time increases. This discrepancy is due to a secondary minor degradation phenomenon, observed only for the less reacted samples, taking place at ≈250 °C (see Figure S11, Supporting Information for derivative TGA curves). FDSC has been performed to analyze the calorimetric response of dry CDs below their *T*_d_^os^. Thanks to the FDSC high sensitivity and its ability to perform heating and cooling scans at a rate of thousands kelvin per second, the technique is the ideal calorimetric tool to investigate metastable states,^[^ ^70^ ^]^ follow fast transformation kinetics,^[^ ^71^ ^]^ and reveal hidden phase transitions even beyond the material degradation temperature, otherwise inaccessible to standard techniques.^[^ ^72^, ^73^ ^]^ FDSC traces recorded at a heating rate of 1000 K/s of dried CDs are shown in Figure 3b. Here, a sharp step in the heat capacity of all samples was highlighted, whose temperature mid‐point (*T*_g_) shifts from 112 °C for CDs_3 to 136 °C for CDs_60, with a monotonic increase with reaction time. This thermal feature was univocally ascribed to the glass transition, confirming the amorphous nature of CDs already emerged from the structural analysis (see Figure 2f). The experimental *T*_g_ values fall in the typical range of polyamides, corroborating the formation of the polyamide networks when β‐Ala and TETA react in microwave. Additionally, the *T*_g_ increasing trend with reaction time (3 to 60 min) supports the hypothesis that longer reaction times produce more rigid nanoparticles.

Noteworthy, the amorphous nature of our CDs enables the study of CDs/suspending medium interactions. Indeed, the variation of *T*_g_ can be exploited to probe whether the suspendant—water in this study—alters the carbon dots’ intrinsic mobility by diffusion through their core, i.e., the plasticization effect. To this aim, the thermal characterization of CDs in wet conditions was implemented with Differential Scanning Calorimetry (DSC). The calorimetric behavior of CDs/water mixtures was recorded at different water weight fractions (*x*_H2O_), and Figure 3c displays the DSC thermograms, acquired during heating at 20 K min^−1^ of CDs_3 (upper panel) and CDs_60 (lower panel) at various *x*_H2O_. Other traces for the overall CDs specimens, comprising both cooling and heating cycles, are shown in Figure S12, Supporting Information). Starting from CDs_3, different thermal behaviors can be distinguished depending on the hydration level. At minor hydrations, *x*_H2O_ ≤ 0.39, a single thermal event is observed, corresponding to the glass transition step of the CDs_3 with absorbed water. Here, water demonstrates to effectively plasticize the dots, causing a gradual decrease of their *T*_g_ from 75 °C (*x*_H2O_ = 0) to −70 °C at higher water content. The absence of additional thermal events supports the hypothesis that water is dispersed within the hydrophilic CDs nanostructure, hence forming a homogeneous mixture. The structural homogeneity of a glass could be depicted by the glass transition width, whereas its broadening is generally ascribed to an increase of the dynamic inhomogeneities and density fluctuations at the nanoscale level.^[^ ^74^, ^75^ ^]^ The glass transition width variation as a function of hydration is shown in Figure S12f (Supporting Information). For CDs_3, similarly to CDs_5 and CDs_10, the glass transition sharpens from ≈25 K (dry) to 11 K by increasing the hydration, revealing that the water insertion inside CDs increases the homogeneity of the glass. Moreover, the intimate interactions between the CDs and water molecules hinder the formation of ice crystals, since no exothermic peaks ascribable to the crystallization and/or melting of water appear in the thermograms. This water type is often referred to as non‐freezable water, since it cannot crystallize even at high supercooling.^[^ ^76^ ^]^ At intermediate hydration, 0.39 < *x*_H2O_ ≤ 0.46, the mixture starts to de‐mix during the heating scan. While no ice is formed during cooling (see Figure S12a lower panel of the Supporting Information), an initial separation occurs on heating scans soon above the glass transition temperature, where a fraction of water undergoes cold‐crystallization (exotherm peaks between −70 and −20 °C, indicated by asterisks in Figure 3c) and, slightly below 0 °C, melts. This kind of water is referred to as freezable water. At higher hydration level, *x*_H2O_ > 0.46, the CDs_3/water mixture separates during cooling, as evidenced by water crystallization occurring between −20 °C and −40 °C and reported in Figure S12a (Supporting Information). During heating, a sudden increase in *T*_g_ from −79 to −52 °C, followed by ice melting between −30 and 0 °C (endothermic peak), is observed.

Some differences in the CDs thermal behavior are highlighted when the synthesis is pushed to more drastic conditions. Considering the most rigid and structured CDs of the series, CDs_60 (lower panel of Figure 3c), the *T*_g_ of the dry CDs is encountered at higher temperature, as already shown by FDSC analysis. The gradual decrease of *T*_g_ from 114 °C up to −44 °C occurs for *x*_H2O_ ≤ 0.35, value at which the *T*_g_ stabilizes up to the maximum hydration reached. The cold‐crystallization of water was hindered for CDs_60, as well as for CDs_10 and CDs_30 (Figure S12c,d, Supporting Information), while the ice melt peak arises already at low hydration levels (0.40 for CDs_60 vs 0.50 for CDs_3). For CDs_60, as well as CDs_30, the glass transition width is stable ≈ 12 K (see Figure S12f, Supporting Information) independently on the hydration level. This result is ascribable to the fact that CDs synthesized with time equal or above 30 min possess more homogeneous structures at the nanometer scale with respect to the less reacted ones. Moreover, water permeation does not alter significantly the structural homogeneity, supporting the calorimetric observation on CDs_60 to have a more compact and less permeable structure.

The plasticization effect of water on the CDs was further clarified by plotting their *T*_g_ as a function of *x*_H2O_, with the resulting trends presented in Figure 3d. At low hydration, the totality of water diffuses inside the bulk CDs and plasticizes the nanoparticles. As a consequence, the *T*_g_ decreases from values way above room temperature (74/114 °C, depending on the CDs) for dry CDs, to values ranging between −44 and −79 °C, depending on the capability of the dots to incorporate water (which in turn is related to their more or less compacted structure). For all specimens, the plasticization effect of water on the dots’ *T*_g_ is predicted by the Fox mixing law at small *x*_H2O_ values:^[^ ^77^ ^]^

(1) $$T_{g}^{-1}=x_{H2O}T_{g,H2O}^{-1}+\left(1-x_{H2O}\right)T_{g,\mathrm{CDs}}^{-1}$$

where *T*_g_ is the glass transition temperature of the hydrated CDs, *T*_g,H2O_ is the glass transition temperature of pure water and *T*_g,CDs_ that of the corresponding dry CDs. The choice of *T*_g, H2O_ = −152 °C (121 K) provided the best fit for all specimens, in line with previous hydration studies on globular proteins and carbohydrate hydrogels.^[^ ^78^ ^]^ It is worth to notice that 121 K is a value significantly lower than the generally accepted water glass transition of 136 K,^[^ ^79^ ^]^ or, more recently, 170 – 200 K.^[^ ^2^ ^]^ However, for the scope of the current study, using the simplest Fox approximation for the *T_g_* prediction of binary mixtures provides a satisfactory fit describing only the CDs “rich” part of the phase diagram. The *T*_g, CDs_ values were taken directly from DSC curves of dry CDs (*T*_g_ of CDs at *x*_H2O_ = 0). At a defined *x*_H2O_ level, specific for each CDs, the *T*_g_ trend falls off the Fox mixing law and stabilizes to a minimum value (*T*_g_^min^). This value corresponds to the maximum plasticization effect, from which the maximum swelling uptake (*x*_H2O_^max^) of CDs can be determined. The latter is extracted from *x*_H2O_ where the glass transition temperature reaches *T*_g_^min^. This point corresponds to where the experimental data deviates from the Fox law prediction, as illustrated for CDs_3 and CDs_60 in Figure 3d. The *T*_g_ stabilization is ascribable to the fact that the inner CDs structure, depending on its rigidity and hydrophilicity, can host a specific amount of water. Above *x*_H2O_^max^, the “exceeding” water molecules start to accumulate outside the CDs, not contributing anymore as plasticizers but suspending the CDs. Noteworthy, *x*_H2O_^max^ monotonously decreases from 0.42 to 0.31 with increasing the CDs reaction time. The swelling capability reduction is in accordance with experimental evidence of the increased rigidity of the carbonaceous core at the expense of hydrophilic functionalities (refer to the aminic reduction in Figure 2e and carboxylate and ammonium disappearing in FTIR spectra of Figure 2d and data retrieved from WAXS of hydrates samples below). The significative parameters of the swelling experiment are summarized in **Table** **1**.

**Table 1:** *T*_g_ of bulk CDs and of hydrated CDs (*T*_g_^min^) at the maximum swelling (*x*_H2O_^m^ ^a^ ^x^) measured by DSC.

Sample	*T*_g,CDs_ ^a^ ^)^ ± 2 °C	*T*_g_^min^ ± 2 °C	*x*_H2O_^max^ ± 0.01
CDs_3	74	−79	0.42
CDs_5	80	−77	0.41
CDs_10	90	−61	0.35
CDs_30	95	−53	0.33
CDs_60	114	−44	0.31

^a)^ *T*_g_ assessed through DSC, at 20 K min^−1^, are 25 – 35 °C lower than those measured by FDSC at 1000 K s^−1^. This discrepancy is in accordance with the kinetic nature of the glass transition, whose transition temperature depend on the perturbation rate applied.^[^ ^80^ ^]^

These plasticization trends are comparable with those reported for globular enzymes such as BSA and lysozyme,^[^ ^3^ ^]^ thus confirming the hypothesized water permeation analogies between polyamide‐based CDs and globular polypeptides.

By increasing the hydration beyond *x*_H2O_^max^, a fraction of water freezes when the mixtures are cooled below 0 °C (see Figure S12, Supporting Information), altering the *T*_g_ of less reacted CDs specimens (*T*_g_ of crystallized mixtures are reported as dot‐in‐symbols in Figure 3d). While the *T*_g_ of more structured samples (CDs_30 and CDs_60) remains stable around *T*_g_^min^, an abrupt increase is evidenced for CDs_3, CDs_5 and, in a minor way, for CDs_10. This phenomenon is already known for hydrogels and xerogels as regulation effect.^[^ ^78^, ^81^ ^]^ Water molecules, indeed, are more mobile and easily extracted from loosely packed nanoparticles, which suffer from major water de‐swelling and anti‐plasticization (water leakage from the nanostructure), as probed by their *T*_g_ increase. The magnitude of the CDs de‐swelling, even if caused by a collateral effect of the experimental setup (the *T*_g_ of hydrated CDs is below the water freezing point), furnishes precious information about the rigidity of the hydrated CDs network. Indeed, it could be hypothesized that CDs suspensions, found at concentrations above *x*_H2O_^max^, could behave in different ways depending on the compactness of their internal structure.

**Figure 4:**
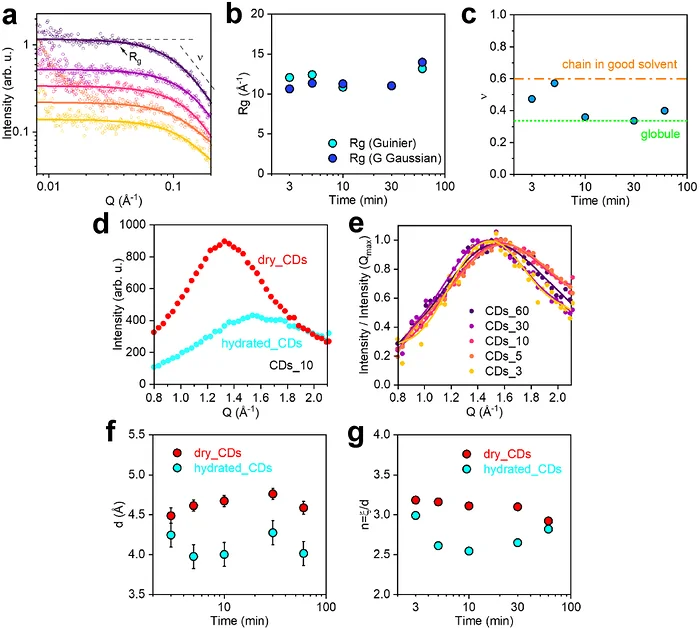
Structural features of the CDs in the presence of water. a) SAXS results of aqueous suspensions (form factors). Data are shifted in the vertical axis for clarity (each by a factor 1.5 with respect to the previous one). Lines are fits of generalized gaussian functions with the radii of gyration displayed in b) and scaling exponents in c). In b), the values of *R*g obtained from Guinier fits are also included. d) Comparison of WAXS results at 25 °C on the sample obtained with 10 min reaction time exposed to ambient humidity (15% *w*_H2O_) and dried. e) Normalized WAXS intensity of the samples corresponding to different reaction times in ambient humidity condition. f) Average intermolecular distance and g) ratio between the short‐range order correlation length and the intermolecular distance as functions of reaction time, for wet and dry samples.

To implement this aspect, the structural features of CD suspensions in water (50 mg mL^−1^ CDs) were investigated by diffraction techniques. Small Angle X‐ray Scattering (SAXS), which allows access to lower Q‐values (larger length scales) than WAXS, was applied to scrutinize the size and conformation of the nano‐objects in suspension. SAXS results on the CDs are shown in **Figure** **4**a. The experimental window accesses well the Guinier regime of the form factors, such that the radius of gyration (*Rg*) can be directly obtained from the initial decay of the intensity. For all reaction times, *Rg* scatter within the range 11 – 14 Å (Figure 4b). These sizes are in good agreement with the CDs diameters within 2 – 4 nm measured from AFM and TEM (Figure 2a). The SAXS curves of samples obtained with different reaction times differ in the high‐Q slope of the scattered intensity, in the so‐called intermediate or fractal regime. This slope is determined by the scaling exponent *ν*, which accounts for the degree of compaction of the nano‐object. For example, for linear polymers *ν* = 0.6 in extended conformation; *ν* = 0.5 in random coil conformation and *ν* = 0.33 in a globule. For the shorter reaction times, the intensity slope is clearly smaller than for the longer ones, reflecting larger values of *ν* and thereby more loosen conformations. The results were fitted by generalized gaussian functions^[^ ^82^ ^]^—usually invoked to describe the form factors of single‐chain nanoparticles—^[^ ^83^, ^84^ ^]^ delivering both parameters *Rg* and *ν*. The resulting values are shown in Figure 3b,c, respectively. The scaling exponents of the CDs obtained with reaction times of 3 and 5 min (*ν*: 0.5 – 0.59) reveal sparse conformations close to random walks and thus these CDs would host large amounts of solvent. On the contrary, the CDs resulting from longer reaction times have close to globular conformations (*ν* ≈ 0.33), within which the presence of water molecules would be scarcer.

The short‐range order of the CDs clearly changes with the inclusion of water molecules, as revealed by WAXS experiments on hydrated samples exposed to room humidity (see CDs water weight fraction at RT and RH in Table S5, Supporting Information). As illustrated in Figure 4d for CDs_10, the structure factor of the hydrated CDs at RT and RH (*x*_H2O_ = 0.15) is much broader than that in the dry state, and the peak position is strongly shifted toward higher Q‐values. This result can be rationalized if new correlations arise between pairs of atoms from water molecules and CDs, with typical distances smaller than the intermolecular ones in the dry state (≈4 Å instead of 4.6 Å, Figure 4f). As estimated from the peak width, upon hydration the correlation length decreases down to 10 Å (Figure S14, Supporting Information, marked as hydrated_CDs), such that the short‐range order prevails over distances smaller than the nearest neighbor (*n* ≈2.5, see Figure 4g).

Ultimately, a preliminary study on moisture absorption kinetics has been performed to investigate the diffusion mechanism of water inside CDs. Dry CDs thin films were exposed to a stable humid environment (at 295 ± 1 K and 58% RH) for different times, to retrieve the water uptake kinetics. The water uptake *M_t_* as a function of exposure time for all CDs specimens was reported in Figure S15a (Supporting Information). Moisture sorption equilibrates for all specimens after 30 – 60 min, reaching a maximum water uptake *M*_e_ (water at equilibrium) of 0.22 – 0.30 g of water per gram of dry CDs, with a gradual decrease from CDs_3 to CDs_60. Considering a 1D vapor diffusion along the CDs film thickness, the fickian diffusion coefficient *D* has been determined following the procedure described in the experimental part. In particular, the *D* determination in Equation (4) utilizes the initial slope of the normalized water uptake (*M_t_/M_e_*) plotted against the square root of time (shown in Figure S15b, Supporting Information). The *D* values, reported in Figure S15c (Supporting Information), were estimated to be 0.8 ± 0.2 × 10^−7^ mm^2^ s^−1^ for all specimens. This value is comparable to those of commercial polyamides, ranging between 1 – 3 × 10^−7^ mm^2^ s^−1^ for nylon 6,6^[^ ^44^, ^85^ ^]^ and 3 × 10^−7^ mm^2^ s^−1^ for PA6,10^[^ ^44^ ^]^ up to reach 6 × 10^−7^ mm^2^ s^−1^ for PA6,^[^ ^86^ ^]^ whereby the diffusion mechanism is likely based on water molecules hopping between neighboring sorption sites, such as amine and amide groups that give hydrogen bonding.

**Figure 5:**
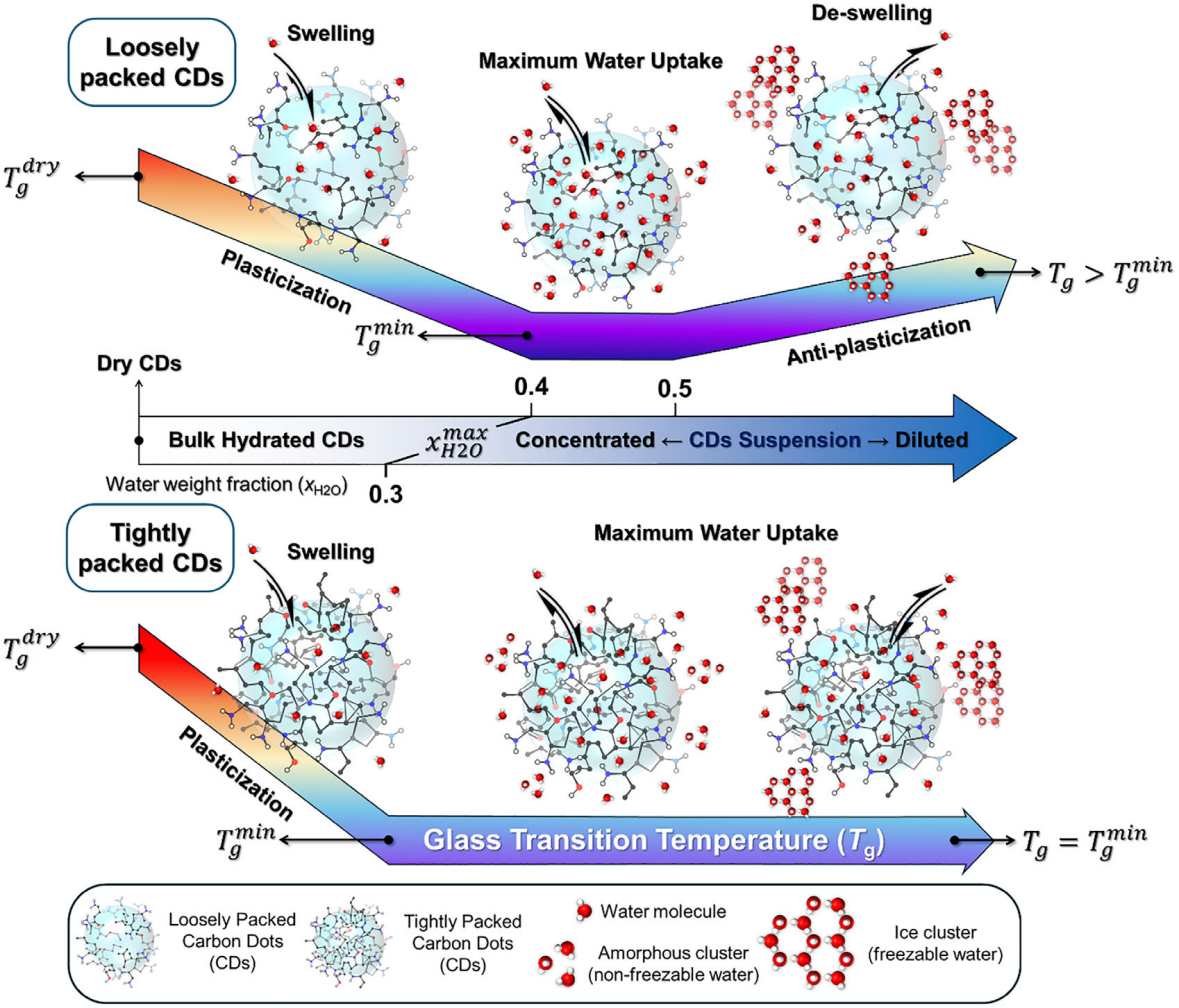
Scheme of the water diffusion mechanism inside amorphous CDs for (upper panel) loosely packed and (lower panel) tightly packed nanoparticles, for different hydration levels (*x*_H2O_). To highlight the de‐swelling/anti‐plasticization phenomenology induced by freezing, the scheme is illustrated at a generic temperature below the water freezing point.

As a final outline, the CDs interaction with water at different hydrations is modeled and shown in **Figure** **5**, in accordance with the detailed characterizations discussed in the current study. The effect of the CDs core structure on the water uptake is represented by differentiating between loosely packed (referring to CDs_3, CDs_5 and CDs_10 specimens, upper panel), and tightly packed CDs (CDs_30 and CDs_60, lower panel).

Three distinct regimes can be identified in the calorimetric profiles (see Figure 3c and Figure S12, Supporting Information), depending on the nanoparticles hydration level, such as i) plasticization by swelling of bulk CDs; ii) maximum water uptake and iii) de‐swelling of suspended CDs induced by freezing.

When a small amount of H_2_O is added to bulk CDs (step i), water spontaneously diffuses into the CDs structures, likely occupying the sites adjacent to the hydrophilic functionalities (both in CDs surfaces and core). The uniform permeation within the nanostructure leads to the swelling of the bulk CDs, which in turn causes the *T*_g_ decrease (plasticizing effect).

By gradually increasing the hydration, an absolute minimum of *T*_g_, namely *T*_g_^min^, is encountered, corresponding to the maximum water uptake capability of CDs (*x*_H2O_^max^). This level of hydration defines the end of the swelling process for the nanoparticles, being the dots saturated. In fact, above *x*_H2O_^max^, the CDs inner core cannot host additional water molecules, reaching the maximum plasticization effect, hence, the *T*_g_ reaches a plateau (*T*_g_ = *T*_g_^min^). In this regime, tightly packed CDs saturate at lower values of *x*_H2O_^max^, undergoing milder swellings and less pronounced *T*_g_ variation. On the other hand, loosely packed CDs reach saturation at higher *x*_H2O_^max^, incorporate more water molecules, hence being more plasticized (lower *T*_g_^min^, see Table 1).

By further increasing the water fraction, bulk hydrated CDs disperse to form aqueous suspensions (step iii) that undergo freezing upon moderate supercooling (between −50 and −20 °C, see DSC cooling traces of Figure S12, Supporting Information).

The structural compactness of CDs dictates their behavior in suspension when stored below the freezing point of water. For loosely packed CDs, the crystallization of the surrounding freezable water can induce de‐swelling. In our opinion, water tends to crystallize preferentially outside the nanoparticles’ architecture. This consideration is based on what was previously observed for water confined in porous silica, in which ice formation was evidenced only inside cylindrical pores with a diameter larger than 2.5 – 2.8 nm.^[^ ^2^, ^87^, ^88^ ^]^ Due to the resembling dimension between the clusters and our CDs, we believe that ice formation inside such polymer‐like nanostructures is unlikely to occur. As external ice crystals grow, they can extract a small fraction of the intercalated, non‐freezable water from the CDs cores. This causes the shrinking of the nanoparticles, leading to an anti‐plasticization effect evidenced by the subsequent *T*_g_ increase. On the other hand, suspensions of tightly packed CDs exhibit greater stability upon water freezing. Their higher core density results in a structure that strongly retains intercalated water, hindering water extraction by the surrounding ice crystals. Consequently, de‐swelling is prevented, and the *T*_g_ of these CDs remains stable at *T*_g_^min^.

## 3 Conclusion

In this work we presented a novel type of carbon dots prepared by β‐Ala and TETA under microwave assisted synthesis in water. For the preparation of the nanoparticles, different conditions were used and the synthetic parameters were optimized following the NMR spectral evolution of the nanomaterials. This approach allowed us to obtain artifact free and pure luminescent carbon dots. Furthermore, different reaction times were used to obtain water soluble CDs with tunable structure packing. We combined the physicochemical characterization of the CDs, which evidenced their polyamide‐based nature, with calorimetric and X‐rays studies in dry, wet state and in aqueous suspension to study the effect of water on the nanoparticles. Our results indicated that the dry CDs, similarly to amorphous materials, possess peculiar glass transition temperatures. The *T*_g_ values were strongly related to the structural compactness of the CDs, as well as the variation of the *T*_g_ with increasing water amount in hydrated conditions. Most interestingly, these results unveiled the capability of water to permeate the CDs inner structure, thus acting as a plasticizer. Water uptake depended upon the core compactness of the carbon dots. In fact, loosely packed CDs, obtained with shorter reaction times, displayed more expanded conformations in aqueous suspensions, retained higher amounts of water and showed a massive shift of the glass transition. On the other hand, longer reaction times allowed us to obtain more compact dots, adopting globular‐like conformation in aqueous suspension, which in turn were able to host lower water quantity and displayed a moderate *T*_g_ shift.

We believe that our results, although expressed for a specific class of amorphous CDs, are of utmost importance to describe the permeability of CDs external shell and core to water, hence providing new tools to understand and better define the elusive nature of carbon dots.

## 4 Experimental Section

### Materials and Methods

β‐Alanine (β‐Ala, 99%) and triethylenetetramine (TETA, 97%) were purchased from Sigma Aldrich and used without any prior purification. Dialysis membrane tubes with a molecular weight cut‐off of 0.5 – 1 kDa were purchased from Spectrum Labs. All the reactions and dialysis were carried out in Milli‐Q water.

### Synthesis of Carbon Dots

To prepare the carbon dots, β‐Ala and TETA were dissolved in Milli‐Q water (300 µL) and stirred at room temperature for 10 min in dark. The mixture was reacted in a CEM Discover 2.0 microwave at 230 °C and 200 W for different times (See Figure S2, Supporting Information for the microwave parameter program). Depending on the reaction time, samples were referred to as “CDs_time”. For instance, CDs obtained with the 30 min reaction are indicated as “CDs_30”. During the microwave treatment, the β‐Ala and TETA mixtures turned from transparent to yellow and then brownish within the first 60 s of irradiation, without the formation of visible insoluble aggregates, even after 60 min of reaction. At the end of the process, the mixtures were cooled down at room temperature, diluted with 4 mL of Milli‐Q water, and filtered through 0.2 µm PTFE syringe filters. Finally, solutions were dialyzed against Milli‐Q water for 3 days (MWCO of 0.5 – 1 kDa) and freeze‐dried to obtain brownish hygroscopic materials. All the CDs were stored at 4 °C before further use. Molar ratios of the reactants for each reaction are reported in Tables S1 and S2 (Supporting Information).

### Nuclear Magnetic Resonance (NMR)

NMR spectra were collected on a Bruker AVANCE III NMR spectrometer (500 MHz). Generally, 5 – 10 mg of material (precursors or CDs) were dissolved in 0.6 mL of D_2_O and analyzed. For proton NMR, 64 scans were recorded using the water suppression modality. The spectra were processed using MestReNova (software version 11.0.2‐18153).

### Atomic Force Microscopy (AFM)

AFM characterization was carried out with a JPK bioAFM system using a tapping mode tip with a frequency of 320 Hz (Bruker TESPA‐V2). A stock solution of nanoparticles was prepared in ultrapure water at 1 mg mL^−1^ concentration, filtrated with 0.1 µm syringe filter and sonicated for 10 min at 25 °C. Then, the stock solution was diluted 1:100 or 1:10000, sonicated and 10 µL were drop casted onto freshly cleaved mica. Images were processed by using WSxM 5.0 develop 3.0 analyzing at least 100 particles for image. Size distributions were analyzed using Microsoft Excel and OriginLab software and plotted using OriginLab.

### Transmission Electron Microscopy (TEM)

TEM images were acquired with a JEOL JEM‐1400 plus microscope with a LaB6 crystal operated at 80 kV. HR‐TEM was performed with a JEOL JEM‐2100F UHR running at 200 kV equipped with a 4 megapixel CMOS camera (TVIPS TemCam‐F216). Samples for TEM and high‐resolution HR‐TEM were prepared by depositing 0.4 µL of CDs (0.1 or 0.01 mg mL^−1^ in water, HPLC grade) on a copper grid coated with a layer of ultrathin carbon film (01824, TED PELLA, INC.), which was freshly UV‐ozone cleaned. Free software ImageJ was used to identify particles (>150 from different window views) and measure their size (largest diameter). Size distributions were analyzed using Microsoft Excel and OriginLab software and plotted using OriginLab.

### Optical Spectroscopy

UV−vis and fluorescence analysis were performed using a Varian Cary 5000 spectrophotometer and an FS5 spectrofluorometer (Edinburgh Instruments). The spectrofluorometer includes an SC‐30 integrating sphere cassette, which was employed to determine the quantum yields (QY %). For both characterizations, carbon dots were dissolved in Milli‐Q water at specific concentration and measured in quartz cuvettes from Hellma (10 mm path length, 0.5 mL). All the experiments were carried out at room temperature. The fluorescence emission profiles reported in arbitrary unit, since QY value was calculated for each sample.

### Fourier Transform Infrared Spectroscopy (FTIR)

FTIR‐ATR (Fourier Transformation‐Attenuated Total Reflection) spectra were recorded using a FTIR spectrometer Invenio‐X (Bruker). Dried samples were analyzed between 4000 – 400 cm^−1^ range, acquiring 64 scans for each spectrum with 4 cm^−1^ resolution.

### Amine Quantification Assay (Kaiser Test)

The amount of amines in the different CDs was calculated using the Kaiser test assay. Briefly, stock solutions of CDs in Milli‐Q water were prepared at a concentration of 10 mg mL^−1^. For each sample, 45 µL of these solutions were transferred into three distinct vials. Subsequently, 75 µL of a phenol solution in ethanol and 100 µL of a potassium cyanide (KCN) solution in pyridine/water were added to both samples and blank solutions. Mixtures were sonicated at room temperature for 2 min to ensure homogeneity. Finally, 75 µL of a ninhydrin solution in ethanol was added. The sample and blank solutions were heated at 120 °C for 5 min, followed by cooling to room temperature for an additional 5 min. Control samples (without CDs) were also performed. Subsequently, the solutions were diluted with 2 mL of a diluting solution (60% ethanol in water), and 750 µL aliquots were transferred into polystyrene cuvettes, where they were further diluted with 2.25 mL of the same diluting solution. Absorbance spectra were acquired using a UV‐vis V730 Jasco spectrophotometer with a scan speed of 400 nm min^−1^ over the range 800 – 400 nm. The absorbance at 570 nm was measured, and the concentration of primary amines was calculated using the following equation:

(2) $$\left(\mu \mathrm{mo}l_{NH_{2}}/g_{\mathrm{CDs}}\right)=\frac{Abs\left(570\mathrm{nm}\right)\cdot 10^{6}}{\varepsilon \left(M^{-1}\cdot {\mathrm{cm}}^{-1}\right)\cdot l\left(\mathrm{cm}\right)\cdot c\left(\mathrm{mg}/\mathrm{mL}\right)}$$

Where *Abs* is the absorbance of the sample at 570 nm, *ɛ* is the molar extinction coefficient of Ruhemann's purple (15000 M^−1^·cm^−1^), *l* is the path length of the cuvette (cm), and *c* is the concentration of the cuvette solution in mg mL^−1^.

### X‐Ray Photoelectron Spectroscopy (XPS)

XPS experiments were performed in a Versaprobe III Physical Electronics (ULVAC) spectrometer with a monochromatic X‐ray source (Aluminum Kα line of 1487 eV), calibrated using the 3d5/2 line of Ag at 368.26 eV. Samples, as powder, were mounted on no‐conductive double‐sided tape. E‐neutralizer and Argon ion neutralizer were used for charge compensation. Z‐alignment was performed for optimal sample height prior to each sample measurement. The elemental quantification was done on survey scan with step energy 0.5 eV, pass energy 224 eV, while high‐resolution regions were acquired with step energy 0.05 eV, pass energy 112 eV, time per step 100 ms (4 sweep for each high‐resolution spectrum). Raw data without further elaboration (Shirley background subtraction) were analyzed by CasaXPS software (2.3.26 PR 1.0).

### Thermogravimetric Analysis (TGA)

All TGA experiments were performed under nitrogen atmosphere (25 mL min^−1^ flow rate) using a TGA Discovery (TA Instruments). The procedure used was composed of a: thermal pretreatment at 120 °C for 5 min, to remove humidity from the samples, followed by a ramp from 40 to 700 °C at 20 °C min^−1^.

### Flash Differential Scanning Calorimetry (FDSC)

FDSC measurements were performed by means of a Mettler Toledo Flash DSC 1, equipped with a Huber TC100 intracooler, and operating in a temperature range between −100 and 450 °C. MultiSTaR UFS1 chip sensors were employed, after calibrating the temperature with the melting point of indium at a heating rate of 1000 K s^−1^. Dry samples of CDs were prepared by depositing, onto the chip's active area, a tiny particle (in the order of 100 ng) of freshly freeze‐dried CDs with a single‐haired brush. Samples were dried in situ by pre‐treating at 180 °C for 1 min, followed by a rapid cooling at 1000 K s^−1^ up to −40 °C. Soon after, five consecutive heating/cooling scans were recorded, with a fixed heating/cooling rate of 1000 K s^−1^ between – 40 and 200 °C. The five curves were averaged before plotting for noise reduction.

### Differential Scanning Calorimetry (DSC)

DSC thermograms were acquired with a TA Instruments Q2000 heat flux calorimeter equipped with liquid nitrogen cooling, allowing to operate between −150 and 600 °C. The oven was purged with dry nitrogen fluxing at 50 mL min^−1^. Temperature and heat flow calibration was performed using Indium Calibration Standard provided by TA Instruments. Low mass TA aluminum pans were used in combination with hermetic lids for samples and reference, to avoid water evaporation during scans. The samples were prepared by depositing ≈1 – 2 mg of freeze‐dried CDs in the pan. Then, samples were placed in a vacuum oven at 120 °C for 30 min and weighted soon after, to retrieve the mass of the dry CDs (*m*_dry_). A defined amount of Milli‐Q water (*m*_H2O_) was added with a micropipette (0.5 – 10 µL) directly onto the dry CDs, to obtain hydrated CDs with a specific water weight fraction (*x*_H2O_ = *m*_H2O_/(*m*_dry_ + *m*_H2O_)). The pans containing hydrated CDs were hermetically sealed and measured at least 12 h after their production, to ensure complete stabilization and hydration homogeneity. An average of ten hydration levels, ranging 0 ≤ *x*_H2O_ ≤ 0.61 were prepared for each CDs specimen. The temperature protocol comprised two heating/cooling cycles between −130 and 40 °C, at a heating/cooling rate of 20 K min^−1^. For lower hydration levels (*x*_H2O_ < 0.15) the scan was performed between −100 and 150 °C.

### Small‐Angle X‐Ray Scattering (SAXS)

SAXS experiments were performed using a Rigaku 3‐pinhole PSAXS‐L instrument operating at 45 kV and 0.88 mA. The system was equipped with a MicroMax‐002+ X‐ray generator, which includes a microfocus sealed‐tube source and an integrated Cu Kα X‐ray generation unit (photon wavelength λ = 1.5406 Å). Both the flight path and the sample chamber were maintained under vacuum conditions. Scattered X‐rays were detected using a Dectris EIGER2 R 1M hybrid photon counting detector, which provides micrometric spatial resolution (75 µm × 75 µm) and a large pixel array (1 million pixels). Azimuthally averaged scattered intensities were recorded as a function of the scattering vector Q, defined as: Q = 4πλ^−1^sin(θ), where 2θ is the scattering angle. Reciprocal space calibration was carried out using silver behenate as the standard. Sample solutions were loaded into boron‐rich capillaries with an outer diameter of 2 mm and a wall thickness of ≈0.01 mm. Samples at a concentration of 50 mg mL^−1^ were measured in transmission geometry, with a sample‐to‐detector distance of 2 meters, covering a Q‐range from 0.008 to 0.20 Å^−1^. A 3 × 3 Detector Scan Profile (DSP) was used, with measurement times of 6000 s per scan. The solvent, Milli‐Q water, was measured under the same conditions and subtracted appropriately from the sample measurements.

### Wide‐Angle X‐Ray Scattering (WAXS)

WAXS experiments were conducted using a Bruker D8 Advance diffractometer equipped with a line‐focus Cu X‐ray tube (operating at 40 kV and 40 mA). The setup featured a parallel beam geometry imposed by a Göbel mirror in the primary beam path, eliminating *K_β_* radiation, and an equatorial axial Soller slit (0.2°) in the secondary beam path. A linear LYNXEYE detector (14.4 × 16 mm active area) was used. Measurements were performed in reflection geometry, varying the 2θ angle from 10° to 30° in 0.05° steps, with a collection time of 10 s per point. The temperature was controlled in an Anton Paar TTK 450 chamber with 1 °C resolution. For each sample corresponding to a given reaction time, first, the ‘*wet*’ sample (previously exposed to ambient humidity at least for 5 h) was measured at 25 °C at ambient conditions, without vacuum. Then, vacuum was applied and the sample was heated to 120 °C for more than 1 h to remove all residual water. Subsequently, under continued vacuum conditions, a second measurement was performed at 25 °C (*dry sample*). The results were binned in groups of 10 to improve the statistics. The background was measured with and without vacuum and subtracted from the measurements. Similar experiments were conducted at 25 °C on the precursors (Figure S13, Supporting Information).

### Moisture Absorption Kinetics

Water vapor absorption kinetics had been determined from time dependent water uptakes, *M_t_(t) = (m_t_ – m_0_) / m_0_*, where *m* are the CDs masses at a time t and at the start, respectively. Measurements were performed in a controlled environment at 295 K and 58% RH (NaBr saturated salt solution). CDs samples were prepared by solution casting in a vial ≈2 – 4 mg of CDs suspended in 0.5 mL of Milli‐Q water. The suspension was evaporated in a vacuum oven at 180 °C for 10 min, obtaining a dry CDs film supported on the bottom of the vial. The estimated average film thicknesses (*h*) ranged between 10 and 25 microns, which were estimated by the formula:

(3) $$h=\frac{V}{A}=\frac{m_{0}}{A\rho }$$

where *A* = 200 mm^2^ is the surface of the film corresponding to the vial bottom area, *V* is the volume determined by the ratio between the dry CDs masses *m_0_* and the density (ρ), arbitrarily fixed at 1 mg mm^−3^, in line with amorphous polyamides.

The diffusion coefficient (*D*) of water was determined by using the simples derivation of the Fick second law for thin films supported on an impermeable substrate.^[^ ^89^ ^]^ Assuming that the diffusion occurs along *h* from the free face to the supported face, *D* has been determined with the simplified formula for the initial diffusion regime:

(4) $$D=\frac{4h^{2}}{\pi }*\frac{M_{t}/M_{e}}{\sqrt{t}}$$

where *M_t_* and *M_e_* are the CDs water uptakes at a time *t* and at the equilibrium, respectively. *M_t_*/*M_e_*/√*t* was determined by the initial slope *M_t_*/*M_e_* plotted against the square root of time, shown in Figure S15b (Supporting Information).

## Conflict of Interest

The authors declare no conflict of interest.

## Supporting information



Supporting Information

## Data Availability

The data that support the findings of this study are available in the supplementary material of this article.
