# Supplementary material for: Water Permeates and Plasticizes Amorphous Carbon Dots: Unraveling the Inner Accessibility of the Nanoparticles by Glass Transition Studies
Source: Adv Mater. 2025 Sep 22;38(51):e10992. doi: 10.1002/adma.202510992 (PMC13568858; doi:10.1002/adma.202510992)
Supplement: Supplementary file 1 [file ADMA-38-e10992-s001.docx]

**Supporting Information**

Water Permeates and Plasticizes Amorphous Carbon Dots: Unraveling the Inner Accessibility of the Nanoparticles by Glass Transition Studies

Elisa Sturabotti,^a^* Valerio Di Lisio,^b,c^* Lucia Cardo,^a^ Alessandro Camilli,^a^ Estela Moretón Alfonsín,^a^ Daniele Cangialosi,^b,c^ Amaia Iturrospe Ibarra,^c^ Arantxa Arbe^c^ and Maurizio Prato^a,d,e^*

^a^ Center for Cooperative Research in Biomaterials (CIC biomaGUNE), Basque Research and Technology Alliance (BRTA), Donostia - San Sebastián 20014, Spain;

^b^ Donostia International Physics Center (DIPC), Paseo Manuel de Lardizabal 4, Donostia - San Sebastián 20018, Spain;

^c^ Centro de Fisica de Materiales (CFM-MPC), CSIC-UPV/EHU, Paseo Manuel de Lardizabal 5, Donostia - San Sebastián E-20018, Spain;

^d^ Ikerbasque, Basque Foundation for Science, Bilbao 48013, Spain;

^e^ Department of Chemical and Pharmaceutical Sciences, INSTM UdR Trieste, University of Trieste, 34127 Trieste, Italy.

Contents

[Preparation of the carbon dots: preliminary studies with β-Ala and TETA	3](#_Toc204939936)

[Characterization of the CDs at different reaction times	4](#_Toc204939937)

[Synthesis	4](#_Toc204939938)

[Atomic Force Microscopy (AFM)	7](#_Toc204939939)

[Transmission Electron Microscopy (TEM)	9](#_Toc204939940)

[Optical properties	12](#_Toc204939941)

[X-ray Photoelectron Spectroscopy (XPS)	13](#_Toc204939942)

[Thermogravimetric Analysis (TGA)	15](#_Toc204939943)

[Differential Scanning Calorimetry (DSC)	16](#_Toc204939944)

[Small Angle X-ray Diffraction (SAXS): results analysis	17](#_Toc204939945)

[Wide Angle X-ray Diffraction (WAXS): results analysis	18](#_Toc204939946)

[WAXS results on precursors:	18](#_Toc204939947)

[Short-range order correlation length from WAXS results:	19](#_Toc204939948)

[Moisture absorption kinetics	20](#_Toc204939949)

[Bibliography	21](#_Toc204939950)

## Preparation of the carbon dots: preliminary studies with β-Ala and TETA

To prepare the carbon dots from β-Ala and TETA, the amount of starting reactants was adjusted by changing their molar ratio (β-Ala:TETA=1:1; 1:0.5; 2:0.5 mol/mol), or selecting different reaction times, between 3 and 60 minutes (Table S1). The reaction temperature was always kept at 230 ºC to guarantee effective condensation, polymerization and carbonization of the molecular species under microwave irradiation, hence obtaining fluorescent nanoparticles.^[1]^ Furthermore, 200 W were used to carry out the reaction in order to reach the desired final temperature in the microwave reactor. By changing the parameters, the reaction between β-Ala and TETA evolved towards more complex structures, as reported in Figure S1. In fact, by comparing CDs_a to CDs_g (the latter referred as CDs_30), the disappearance of sharp signals with the increase of reaction times is evident (from 3, to 5 to 30 minutes of microwave treatment).

**Table S1.** Reaction parameters for the attempted synthesis of CDs. All the reactions were carried out at 230 °C and at 200 W in Milli-Q water.

| **Reaction** | **β-Ala (mmol)** | **TETA (mmol)** | **COOH:NH_x_^*^** | **V (µL)** | **t (min)** |
| --- | --- | --- | --- | --- | --- |
| **CDs_a** | 1 | 0.5 | 1:3 | 300 | 3 |
| **CDs_b** | 1 | 0.5 | 1:3 | 300 | 5 |
| **CDs_c** | 1 | 1 | 1:5 | 300 | 5 |
| **CDs_d** | 1 | 1 | 1:5 | 150 | 5 |
| **CDs_e** | 1 | 0.5 | 1:3 | 300 | 10 |
| **CDs_f** | 1 | 0.5 | 1:3 | 300 | 30 |
| **CDs_g (CDs_30)** | 2 | 0.5 | 2:4 | 300 | 30 |

X = 1 or 2

**Figure S1.** ^1^H NMR of carbon dots obtained following parameters reported in Table S1. All the samples were dissolved in D_2_O (~ 5 mg in 0.6 mL) and spectra were recorded at 500 MHz. To avoid residual H_2_O signal, spectra in water suppression were carried out.

## Characterization of the CDs at different reaction times

### Synthesis

**Figure S2.** Microwave reactor parameters diagram during the synthesis of CDs_30.

**Table S2.** Reaction details for CDs obtained with β-Ala:TETA molar ratio of 2:0.5. All the reactions were carried out at 230 °C and at 200 W in Milli-Q water.

| **Sample** | **Time (min)** | **Mass (mg)** | **Mass yield (%)^a^** |
| --- | --- | --- | --- |
| **CDs_3** | 3 | 4.1 ± 1.2 | 1.6 |
| **CDs_5** | 5 | 5.7 ± 2.7 | 2.2 |
| **CDs_10** | 10 | 15.5 ± 1.5 | 6.2 |
| **CDs_30** | 30 | 40.2 ± 3.6 | 15.9 |
| **CDs_60^b^** | 60 | 21.4 ± 1.1 | 8.5 |

^a^ Calculated with respect to the amount of starting materials.

^b^ For the preparation of CDs_60 two consecutive cycles of 30 minutes were used.

**Figure S3.** ^1^H NMR of CDs obtained using β-Ala:TETA molar ratio of 2:0.5 and changing reaction time (from 3 to 60 minutes). All the samples were dissolved in D_2_O (~ 5 mg in 0.6 mL) and spectra were recorded at 500 MHz. To avoid residual H_2_O signal, spectra were carried out using water suppression.

**Figure S4.** Carbon dots obtained at different reaction times in water at 2 mg mL^-1^. From left to right: CDs_3, CDs_5, CDs_10, CDs_30 and CDs_60.

### Atomic Force Microscopy (AFM)

**b**

**c**

**d**

**e**

**a**

**Figure S5.** AFM images of the synthesized CDs. a) CDs_3. b) CDs_5. c) CDs_10. d) CDs_30 and e) CDs_60.

**Figure S6.** Size (height) distributions for the complete set of CDs retrieved by AFM analysis.

### Transmission Electron Microscopy (TEM)

**a**

**b**

**c**

**d**

**e**

**Figure S7.** TEM micrograph of CDs acquired at different magnifications. a) CDs_3. b) CDs_5. c) CDs_10. d) CDs_30 and e) CDs_60.

**Figure S8.** Size (diameter) distributions for the complete set of CDs retrieved by TEM analysis.

### Optical properties

**Figure S9.** Fluorescence profiles of CDs obtained after 3, 5, 10, 30 and 60 minutes.

**Table S3.** Quantum yield (QY %) values measured at 350 nm for each type of CDs.

| **Sample** | **QY %** |
| --- | --- |
| **CDs_3** | 16.2 |
| **CDs_5** | 15.8 |
| **CDs_10** | 22.4 |
| **CDs_30** | 23.4 |
| **CDs_60** | 19.2 |

### X-ray Photoelectron Spectroscopy (XPS)

**Figure S10.** Survey and high resolution (C 1s, N 1s and O 1s) XPS spectra for each type of CDs.

**Table S4.** XPS fitted components for C 1s, N1s and O 1s for each CDs.

|  | **CDs_3** | | **CDs_5** | | **CDs_10** | | **CDs_30** | | **CDs_60** | |
| --- | --- | --- | --- | --- | --- | --- | --- | --- | --- | --- |
| **C1s** | Position  (eV) | Area (%) | Position  (eV) | Area (%) | Position  (eV) | Area (%) | Position  (eV) | Area (%) | Position  (eV) | Area (%) |
| **C=C/C-C** | 284.8 | 48.2 | 284.8 | 47.9 | 284.8 | 54.4 | 284.8 | 63.6 | 284.8 | 60.4 |
| **C-O/C-N** | 285.9 | 35.1 | 285.8 | 34.8 | 285.8 | 28.0 | 285.9 | 20.8 | 285.7 | 23.7 |
| **C=O/C=N** | 287.6 | 15.2 | 287.7 | 15.8 | 287.5 | 12.6 | 287.2 | 9.2 | 287.2 | 8.6 |
| **O-C=O** | 288.8 | 1.5 | 288.9 | 1.4 | 288.5 | 4.9 | 288.7 | 6.4 | 288.7 | 7.3 |

|  | **CDs_3** | | **CDs_5** | | **CDs_10** | | **CDs_30** | | **CDs_60** | |
| --- | --- | --- | --- | --- | --- | --- | --- | --- | --- | --- |
| **N1s** | Position  (eV) | Area (%) | Position  (eV) | Area (%) | Position  (eV) | Area (%) | Position  (eV) | Area (%) | Position  (eV) | Area (%) |
| **R-NH** | 399.4 | 75.8 | 399.4 | 81.1 | 399.4 | 85.7 | 399.5 | 93.4 | 399.6 | 100 |
| **-NH_3_^+^** | 400.8 | 24.2 | 400.8 | 18.9 | 400.8 | 14.3 | 401.6 | 6.6 | - | - |

|  | **CDs_3** | | **CDs_5** | | **CDs_10** | | **CDs_30** | | **CDs_60** | |
| --- | --- | --- | --- | --- | --- | --- | --- | --- | --- | --- |
| **O1s** | Position  (eV) | Area (%) | Position  (eV) | Area (%) | Position  (eV) | Area (%) | Position  (eV) | Area (%) | Position  (eV) | Area (%) |
| **C=O** | 530.9 | 61.7 | 530.9 | 65.5 | 531.1 | 74.0 | 531.4 | 57.7 | 531.6 | 62.8 |
| **C-O** | 532.1 | 32.3 | 532.1 | 27.1 | 533.1 | 26.0 | 533.2 | 42.3 | 533.3 | 37.2 |
| **C-OH** | 533.4 | 6.0 | 533.3 | 7.4 | - | - | - | - | - | - |

### Thermogravimetric Analysis (TGA)

TGA is a helpful technique to define the operative window for CDs, *i.e.*, the region in which nanoparticles are thermally stable, and it can support the choice of precursors, by selecting the optimal temperature to react them (generally before their degradation). Thermogravimetric curves, displayed in Figure 3a, revealed a similar thermal stability for all the analyzed CDs. TGA curves are comprised of two degradation phenomena. A first weight drop occurs at about 245 °C and is evident for samples CDs_3 and CDs_5, while the second abrupt and broad mass loss at 390 °C is related to overall degradation of the nanoparticles. Degradation temperatures are measured as the temperature at the minimum of the weight loss derivative with temperature curve (derivative weight loss percentage DTGA), in Figure S11 below.

**Figure S11.** DTGA curves of the CDs series.

### Differential Scanning Calorimetry (DSC)

**Figure S12.** DSC traces of (a) CDs_3, (b) CDs_5, (c) CDs_10, (d) CDs_30, (e) CDs_60 with different water weight fraction *x*_H2O_, during heating (upper panels) and cooling (lower panels) cycles at 20 K min^-1^. (f) Glass transition width as a function of *x*_H2O_. Solid lines are a guide for the eyes to appreciate the *T*_g_ width trend for loosely packed (CDs_3, CDs_5, CDs_10) and tightly packed (CDs_30, CDs_60) CDs.

### Small Angle X-ray Diffraction (SAXS): results analysis

At low *Q*-values, the fit of the initial decay of the intensity to the Guinier approximation:

$I\left( Q \right)=Aexp(-\frac{1}{3}Q^{2}R_{g}^{2})$	Eq. S1

allows obtaining model-free information about the dimensions of the particles (*R_g_*).

The scattered intensity of nano-objects in dilute solution is proportional to their form factor *P*(*Q*). The results were fitted by using the Sasfit program.^[2,3]^ For the form factor *P*(*Q*) a generalized Gaussian coil function^[4]^ was assumed:

$P\left( Q \right)=\frac{1}{\upsilon U^{\frac{1}{2\upsilon}}}\gamma\left( \frac{1}{2\upsilon},U \right)-\frac{1}{\upsilon U^{\frac{1}{\upsilon}}}\gamma\left( \frac{1}{\upsilon},U \right)$	Eq. S2

Where:

$U=\frac{(2\upsilon+1)(2\upsilon+2)}{6}Q^{2}R_{g}^{2}$	Eq. S3

$\gamma\left( a,x \right)=\int_{0}^{x} t^{\alpha-1}e^{-t}dt$	Eq. S4

### Wide Angle X-ray Diffraction (WAXS): results analysis

**Scherrer Equation:**

The Scherrer equation:^[5]^

$D=\frac{k\lambda}{FWHM\left( 2\theta\right)cos(\theta)}$	Eq. S5

Describes the broadening of a peak at a particular diffraction angle, as it relates the crystalline domain size (*D*) to the width of the peak at half of its height. The Scherrer constant, *κ*, is typically considered to be 0.9 but can vary with the morphology of the crystalline domains. To apply this equation, the *θ* angle in the calculation of FWHM(2θ) and cos(θ) must be expressed in radians.

### WAXS results on precursors:

**a**

**b**

**Figure S13.** WAXS results on β-Ala (a) and TETA (b) at 25°C. The pattern of TETA revealed its liquid state at RT, while β-Alanine showed a clear crystalline nature.

For TETA, *d* = 4.25 Å, *ξ* = 14 Å and *n* = *ξ*/*d* = 3.25.

### Short-range order correlation length from WAXS results:

**Figure S14.** Short-range order correlation length *ξ*=*4π*/FWHM as function of reaction time deduced from the WAXS peak width for the dry and hydrated samples.

**Table S5.** Water weight fraction (*x*_H2O_) of the CDs stored at ambient humidity (RH) and room temperature (RT).

| **Sample** | ***x*_H2O_ at RH** |
| --- | --- |
| **CDs_3** | 0.22 |
| **CDs_5** | 0.21 |
| **CDs_10** | 0.15 |
| **CDs_30** | 0.12 |
| **CDs_60** | 0.11 |

### Moisture absorption kinetic

**Figure S15.** Moisture absorption kinetics measurements for CDs specimens. (a) CDs water uptakes *M*_t_ as a function of time. The curves are vertically shifted by 0.1 *g*_H2O_/*g*_CDs_ between each other to increase clarity. *M*_e_ is the water uptake at equilibrium. (b) *M*_t_/*M*_e_ ratio as a function of the square root of time. The slope was used to determine the (c) fickian diffusion coefficient *D*.

## Bibliography

[1]	T. V. De Medeiros, J. Manioudakis, F. Noun, J.R. Macairan, F. Victoria, R. Naccache, Microwave-assisted synthesis of carbon dots and their applications, J Mater Chem C Mater 7 (2019) 7175–7195. https://doi.org/10.1039/c9tc01640f.

[2]	I. Breßler, J. Kohlbrecher, A.F. Thünemann, SASfit: a tool for small-angle scattering data analysis using a library of analytical expressions, Applied Crystallography 48 (2015) 1587–1598. https://doi.org/10.1107/S1600576715016544

[3]	J. Kohlbrecher, I. Breßler, Updates in SASfit for fitting analytical expressions and numerical models to small-angle scattering patterns, Applied Crystallography 55 (2022) 1677–1688. https://doi.org/10.1107/S1600576722009037

[4]	B. Hammouda, Small‐angle scattering from branched polymers, Macromol Theory Simul 21 (2012) 372–381. https://doi.org/10.1002/mats.201100111

[5]	P. Scherrer, Bestimmung der Grosse und inneren Struktur von Kolloidteilchen mittels Rontgenstrahlen, Nach Ges Wiss Gottingen 2 (1918) 8–100.
